# Characterization of chronic and acute ESA hyporesponse: a retrospective cohort study of hemodialysis patients

**DOI:** 10.1186/s12882-015-0138-x

**Published:** 2015-08-18

**Authors:** Scott P. Sibbel, Carol E. Koro, Steven M. Brunelli, Alexander R. Cobitz

**Affiliations:** DaVita Clinical Research, Minneapolis, MN USA; GlaxoSmithKline, 1250 South Collegeville Road, Collegeville, PA 19426 USA

**Keywords:** ESA hyporesponse, Erythropoietin, Hemoglobin, Hemodialysis

## Abstract

**Background:**

Some patients with chronic kidney disease do not respond adequately to erythropoiesis-stimulating agent (ESA) treatment; these patients are referred to as ESA hyporesponders. There is no widely accepted contemporary definition for chronic ESA hyporesponse. The study objective was to propose and validate an operational definition for chronic ESA hyporesponse.

**Methods:**

This was a retrospective cohort study using electronic health care records. Participants were anemic hemodialysis patients treated during February 2012 and were followed for 15 months. Patients’ ESA response (responders) or lack of response (chronic and acute hyporesponders) based on longitudinal patterns of ESA dose and hemoglobin level was assessed. Persistence of hyporesponse, longitudinal iron measures, transfusion rates, and mortality rates were analyzed. Frequency of blood transfusions (monthly) and death rates (quarterly) were calculated. Log normalized serum ferritin concentration was analyzed.

**Results:**

Of 97,677 eligible patients, 6632 had acute hyporesponsiveness (ESA responsiveness restituted in ≤ 4 months) and 3086 had chronic hyporesponsiveness (lack of ESA response for > 4 months). Over months 1–4 among chronic hyporesponders, mean serum ferritin (722–785 ng/mL) and transferrin saturation (TSAT; 26.76 %-27.08 %) were constant, while acute hyporesponsive patients experienced increased ferritin (654-760 ng/mL) and TSAT (25.71–30.88 %) levels. Compared to ESA responders (0.19–0.30 %), chronic hyporesponders were transfused 7-times (1.20–2.17 %) more frequently over follow-up. Quarterly mortality was greatest in chronic ESA hyporesponders (2.98–5.48 %), followed by acute ESA hyporesponders (2.17–3.30 %) and ESA responders (1.43–2.49 %). With consistence over the study, chronic hyporesponders died more frequently than patients in the other study cohorts.

**Conclusions:**

Findings indicate that 4 months of continuous ESA hyporesponsiveness can be used to differentiate acute from chronic hyporesponsiveness. This definition of chronic hyporesponsiveness is supported by outcome data showing higher mortality and transfusion rates in chronic hyporesponders compared to acute hyporesponders.

**Electronic supplementary material:**

The online version of this article (doi:10.1186/s12882-015-0138-x) contains supplementary material, which is available to authorized users.

## Background

Anemia is a common complication in patients with chronic kidney disease and end-stage renal disease (ESRD). It is estimated that at any time approximately 85 % of hemodialysis patients in the United States are treated with erythropoiesis-stimulating agents (ESAs) [1] such as epoetin alfa, which are recombinant human biologic products of the peptide hormone erythropoietin. Erythropoietin and ESAs enhance red blood cell production in the bone marrow, subsequently increasing hemoglobin thereby avoiding transfusion in treated patients. ESA dose is titrated to target a desired hemoglobin concentration [2]. The doses of ESA necessary to achieve a specific hemoglobin concentration can fluctuate with patient circumstances such as the occurrence of infections, illnesses and hospitalizations, and changes in iron stores [3–5].

ESA resistance, or hyporesponsiveness, is a state in which hemoglobin response is less than typically anticipated for a given dose of ESA [6–8]. ESA hyporesponsiveness is associated with a higher risk of all-cause mortality [6, 9, 10], and may be acute (i.e., occurs for some duration and then remits) or chronic (i.e., persists on a permanent or semi-permanent basis) [11]. Escalation of ESA dose in hyporesponders does little to ameliorate anemia [12]. In fact, data suggest that high levels of ESAs used in these patients may be associated with adverse clinical outcomes such as stroke and vascular access thrombosis [2, 13, 14]. Nonetheless, high-dose ESAs are commonly administered to hyporesponders at great cost to the health care system [1] and to the possible detriment of patients.

There is no consensus, or definition, of what constitutes ESA hyporesponsiveness in general or chronic ESA hyporesponsiveness in particular [15]. In the past, ESA hyporesponsiveness was defined as requirement for a requisite minimum dose of ESA in order to maintain hemoglobin in the target range (i.e., 10–12 g/dL) [16] or failure to reach the target range despite such a dose [17, 18]. However, revised anemia guidelines from Kidney Disease Improving Global Outcomes (KDIGO) no longer specify a lower hemoglobin target bound, complicating application of such definitions [15]. Even prior to guideline changes, definitions of chronic ESA hyporesponsiveness were heterogeneous [19]. A lack of definitional consistency impedes interpretation of the literature and creates barriers to subsequent research aimed at characterizing the burden of, and potential treatments for, chronic ESA hyporesponsiveness.

The objective of the current study was to develop and validate an empiric definition of chronic ESA hyporesponsiveness. A prerequisite was to first create baseline criteria for ESA hyporesponsiveness that are applicable in contemporary nephrology practice. Subsequently, these criteria for ESA hyporesponsiveness were applied over time to determined whether lack of ESA response was acute (transient) or chronic (persistent).

## Methods

### Setting, patients, and data source

We conducted a retrospective cohort study of a clinical database containing the electronic medical records of approximately 170,000 dialysis patients treated at over 2000 US dialysis facilities annually within a large dialysis organization. Since this was a retrospective, observational study, informed patient consent was not required and the analysis was exempt from institutional review board or ethics committee approval. An exemption of consent was granted by Quorum Investigational Review Board based in Seattle due to low/minimal risk for disclosure of personal health information. The database captures patient demographics, injectable medications (e.g., ESA, intravenous iron, and vitamin D), clinical laboratory values, dialysis treatment records, and all comorbidities available to the dialysis units. Patient data are anonymized. Our study included monthly patient-level data for a 15-month period between 01 February 2012 and 30 April 2013. Patients included in the analysis were ≥ 18-years old as of 01 February 2012 and had been receiving thrice weekly in-center hemodialysis prior to 01 February 2012. Patients were excluded if they were receiving peritoneal dialysis, home hemodialysis, or nocturnal hemodialysis; had a documented history of cancer; or were currently receiving cancer treatment. To minimize the impact of acute illness on hyporesponse measures, patients were also excluded if they were hospitalized during January 2012, the month prior to baseline assessments.

### Operational baseline criteria for ESA hyporesponse

To inform baseline criteria for ESA hyporesponsiveness we examined data from month 1 of the study (baseline month of February 2012). We calculated individual patients’ mean hemoglobin concentrations and per administration ESA doses (cumulative monthly dose divided by the number of scheduled in-center hemodialysis treatments). These values were cross tabulated to characterize the distribution of patients’ hemoglobin-by-ESA dose strata. Patients may have missed dialysis sessions for a variety of reasons, however, these sessions are not included in the per session ESA dose calculation. We identified a series of contiguous strata that were logically consistent with ESA hyporesponsiveness. Each specified stratum was characterized by ESA dose > 72^nd^ percentile for the cohort (corresponding to > 4000 erythropoietin U/treatment). Increasingly restrictive thresholds for ESA dose were required for strata characterized by higher hemoglobin: e.g., for hemoglobin > 9.5 to 10 g/dL ESA dose threshold was > 6000 U/treatment (i.e., > 84^th^ percentile); for hemoglobin > 10 to 10.5 g/dL ESA dose threshold was > 8000 U/treatment (i.e., > 89^th^ percentile); for hemoglobin > 10.5 to 11 g/dL ESA dose threshold was > 10,000 U/treatment (i.e., > 93^rd^ percentile), etc. Patients identified using this hemoglobin-by-ESA dose strata were identified as ESA hyporesponders. The type of the hyporesponse (acute vs. chronic) was determined by considering patients’ hemoglobin-by-ESA dose strata over time.

### Longitudinal definitions of ESA hyporesponse

The definition of chronic ESA hyporesponsiveness was based on the consideration: *after how many months of consecutive hyporesponsiveness is it unlikely that patients will subsequently regain ESA responsiveness*? For this study, we considered all patients who met criteria for ESA hyporesponse at baseline and conducted a Kaplan Meier analysis to characterize time to restitution of ESA responsiveness (i.e., the first month in which each patient no longer met the baseline criteria for ESA hyporesponse). We used these results to identify the month that the Kaplan Meier curve flattened, at which time restitution of ESA responsiveness was unlikely, and thereby defining a minimum requirement for chronic hyporesponsiveness. It should be noted that this definition is consistent with previous evidence generated at the time of data analysis by Gilbertson et al. 2013. Patients who were ESA hyporesponse but in whom hyporesponse did not persist for this requisite duration were categorized as acute hyporesponders.

Patients were characterized into 3 mutually exclusive categories for analysis: 1) ESA responders, who did not meet criteria for ESA hyporesponse in month 1; 2) acute ESA hyporesponders, who met criteria for ESA hyporesponse in month 1 of study but in whom ESA hyporesponse did not persist long enough thereafter to qualify as chronically ESA hyporesponsive; 3) chronic ESA hyporesponders, who met criteria for ESA hyporesponse in month 1 of study and in whom ESA hyporesponse lasted long enough to qualify as persistent according to Kaplan Meier analysis.

### Comparisons among chronic ESA hyporesponders, acute ESA hyporesponders, and ESA responders

Among these 3 groups we compared baseline demographic, comorbidity, and laboratory characteristics. Ferritin and transferrin saturation (TSAT) levels were compared over months 1 through 4 of study (i.e., the period of time over which distinctions between acute and chronic hyporesponse were made). These iron indices were studied to examine whether iron stores differed between acute and chronic ESA hyporesponders and may have contributed to ESA hyporesponse. Transfusion rates were compared among study groups over months 1 through 7. Transfusion data, which were obtained by a third-party vendor from inpatient records, were not available after August 2013. Finally, quarterly mortality rates were compared among study groups; to avoid immortal time bias, mortality was considered beginning in month 5 (i.e., after the distinction between acute and chronic ESA hyporesponse was made).

### Statistical methods

Categorical variables were characterized as counts and proportions. Continuous variables were characterized as means, standard deviations, medians, and ranges.

Kaplan Meier analysis was conducted to determine the time to restitution for ESA responsiveness among patients who met criteria for ESA hyporesponse in month 1.

Linear mixed models were used to compare mean ferritin, mean TSAT, and transfusion rates. Ferritin values were skewed and therefore were log transformed; results were then presented on the actual scale. Transfusions were categorized as dichotomous (yes, no) for each patient-month based on whether the patient received any red blood cell transfusions during the month; results were expressed as the proportion of patients transfused. Mortality was analyzed by life-table methods using quarterly intervals beginning in study month 5. For all analyses, patients were censored at the time of transfer of care, kidney transplant, dialysis modality change, withdrawal from dialysis, or death.

## Results

A total of 97,677 patients were identified as eligible for the analysis. Of those, 10,825 (11.08 %) patients met criteria for ESA hyporesponse in month 1 (baseline, February 2012) of the study (Table 1). For internal validation purposes, we performed a similar analysis for the month of July 2011 (a time during which ESA dosing in ESRD was substantially different [20–22]) and observed a similar proportion of patients who met the criteria for ESA hyporesponse (11.56 %) (Additional file 1: Table S1).

**Table 1:** Baseline distribution of ESA dose by hemoglobin concentration: February 2012

		ESA Dose Per Session (IU) Range										
		Missing	0- 2000	>2000-4000	>4000-6000	>6000-8000	>8000-10,000	>10,000-12,000	>12,000-14,000	>14,000-16,000	>16,000	
		% (n)	% (n)	% (n)	% (n)	% (n)	% (n)	% (n)	% (n)	% (n)	% (n)	% (n)
Hemoglobin Range (g/dL)	Missing	0.18 (174)	0.12 (113)	0.13 (128)	0.12 (116)	0.06 (56)	0.06 (59)	0.03 (29)	0.02 (15)	0.04 (38)	0.01 (14)	0.76 (742)
	<8	0.02 (18)	0.02 (16)	0.04 (36)	**0.05 (49)**	**0.05 (47)**	**0.05 (49)**	**0.04 (43)**	**0.05 (53)**	**0.14 (137)**	**0.11 (109)**	0.57 (557)
	>8-8.5	0.02 (21)	0.04 (36)	0.13 (128)	**0.13 (128)**	**0.10 (94)**	**0.09 (92)**	**0.06 (57)**	**0.08 (82)**	**0.15 (151)**	**0.11 (111)**	0.92 (900)
	>8.5-9	0.03 (34)	0.16 (156)	0.37 (366)	**0.34 (336)**	**0.23 (226)**	**0.17 (170)**	**0.16 (159)**	**0.17 (163)**	**0.23 (225)**	**0.11 (111)**	1.99 (1946)
	>9-9.5	0.06 (59)	0.57 (552)	1.15 (1124)	**0.82 (802)**	**0.46 (452)**	**0.33 (327)**	**0.26 (253)**	**0.25 (242)**	**0.31 (299)**	**0.15 (149)**	4.36 (4259)
	>9.5-10	0.11 (109)	1.92 (1872)	2.85 (2780)	1.64 (1601)	**0.86 (842)**	**0.61 (596)**	**0.34 (330)**	**0.36 (355)**	**0.34 (351)**	**0.15 (160)**	9.21 (8996)
	>10-10.5	0.36 (352)	5.44 (5310)	5.09 (4975)	2.56 (2504)	1.18 (1155)	**0.88 (856)**	**0.49 (477)**	**0.33 (326)**	**0.32 (314)**	**0.14 (135)**	16.80 (16,404)
	>10.5-11	1.0 (979)	10.06 (9828)	6.19 (6050)	2.81 (2744)	1.36 (1327)	0.89 (866)	**0.34 (335)**	**0.22 (212)**	**0.16 (158)**	**0.07 (73)**	23.11 (22,572)
	>11-11.5	1.94 (1892)	10.25 (10,010)	4.34 (4236)	1.96 (1918)	0.95 (930)	0.52 (505)	0.19 (185)	**0.08 (78)**	**0.04 (36)**	**0.05 (48)**	20.31 (19,838)
	>11.5-12	2.88 (2811)	5.24 (5115)	1.91 (1868)	0.79 (733)	0.35 (343)	0.14 (137)	0.07(68)	0.02 (19)	**0.01 (12)**	**0.02 (15)**	11.43 (11,161)
	>12	6.75 (6593)	1.98 (1936)	0.97 (951)	0.48 (469)	0.18 (180)	0.09 (85)	0.04 (43)	0.01 (16)	0.01 (12)	0.10 (7)	10.54 (10,292)
		13.35 (13,042)	35.78 (34,944)	23.18 (22,642)	11.71 (11,440)	5.79 (5652)	3.83 (3742)	2.03 (1979)	1.60 (1561)	1.77 (1733)	0.95 (932)	100 (97,677)

Bold cells indicate domains consistent with the baseline definition of ESA hyporesponse

**Fig. 1:**
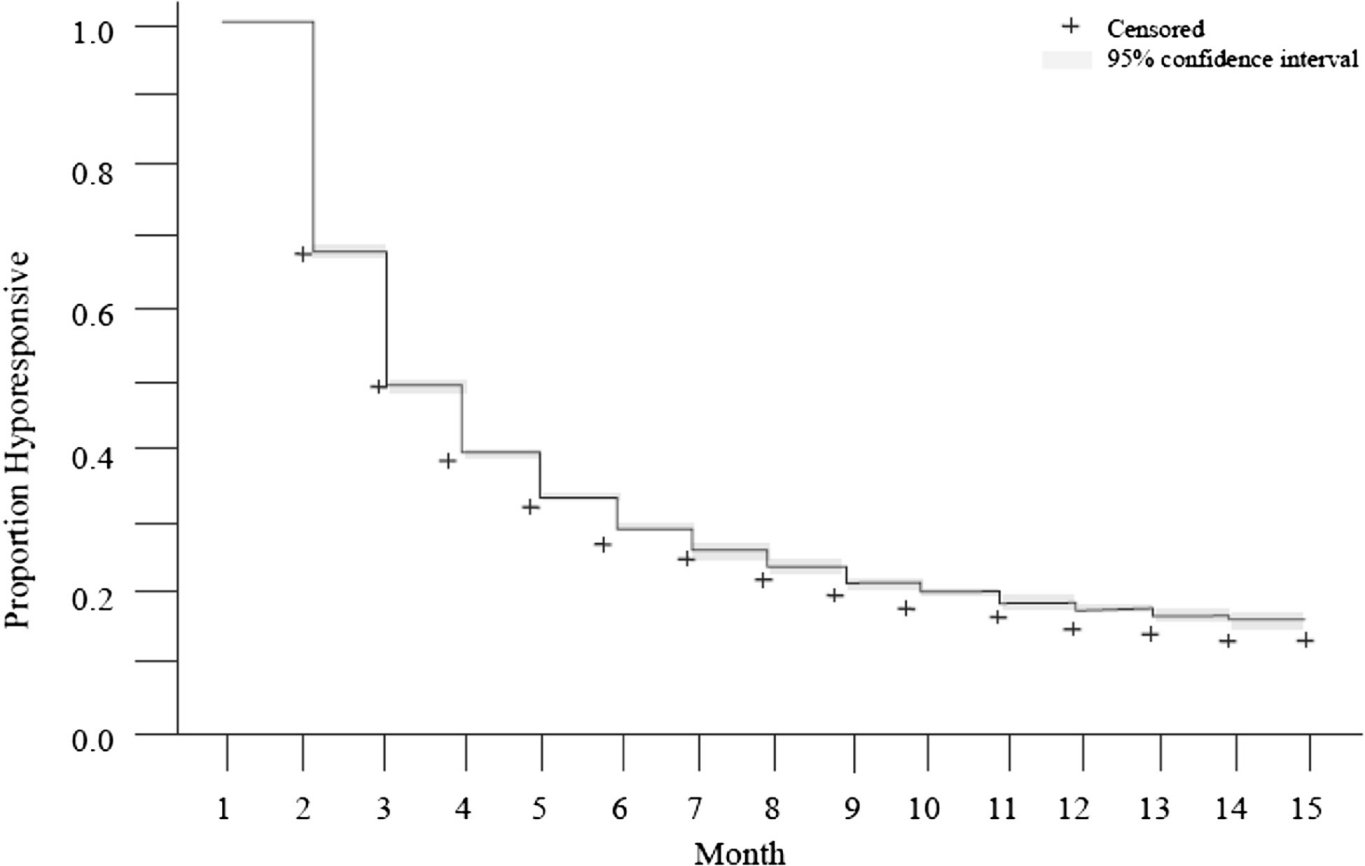
Shown is a Kaplan Meier analysis illustrating hemodialysis patients’ return to ESA responsiveness according to ESA dose by hemoglobin concentrations for all patients who were hyporesponsive at month 1 (baseline). ^+^Indicates censoring during month for transfer of care, kidney transplant, dialysis modality change, withdrawal from dialysis, or death

Figure 1 shows the Kaplan Meier cumulative survival plot. It illustrates the time to restitution of ESA response in months. For the majority of patients (61.3 %), ESA responsiveness was regained by month 4; thereafter, the curve reached a plateau. Based on this finding, acute ESA hyporesponse was defined as hyporesponse lasting < 4 months, and chronic ESA hyporesponse was defined as hyporesponse persisting ≥ 4 months.

Of the 10,825 baseline hyporesponders 1107 were censored by the end of month 4 (127 were lost to follow up, 55 received kidney transplants, 2 had dialysis modality changes, 500 had transfer of care, and 423 died). Of the remaining 9718 baseline hyporesponders, 6632 (68.2 %) were acute hyporesponders and 3086 (31.8 %) were chronic hyporesponders. Of 97,677 patients available at baseline, 81,504 ESA responders were still available for study through the end of month 4 and available for subsequent analyses.

Baseline demographic characteristics for chronic hyporesponders, acute hyporesponders, and ESA responsive patients are shown in Table 2 (continuous variables) and Table 3 (categorical variables). The mean age was lower for chronic hyporesponders (58.8 years) versus acute hyporesponders (60.1 years) versus responders (62.4 years). Dialysis vintage varied between 3.8 and 4.1 years across all 3 groups. Chronic ESA hyporesponders had higher mean body mass index (29.1 kg/m [2]) than the other 2 groups. At baseline, ESA responders had higher mean hemoglobin (11.0 g/dL) and TSAT (31.8 %) than either acute or chronic hyporesponders, who were similar to one another. Minor differences in median serum ferritin were observed among study groups at baseline: 748, 731, and 757 ng/mL for chronic hyporesponders, acute hyporesponders, and responders, respectively. Sex and racial mix were similar across all 3 groups. Chronic (21.7 %) and acute (23.8 %) hyporesponders were more commonly dialyzed via a central venous catheter than ESA responders (14.0 %).

**Table 2:** Comparison of baseline characteristics among ESA responders, acute hyporesponders, and chronic hyporesponders: continuous variables

	Chronic hyporesponders (*n* = 3086)			Acute hyporesponders (*n* = 6632)			ESA responders (*n* = 81,504)			*P* value
	Mean	SD	Median	Mean	SD	Median	Mean	SD	Median	
Age (years)	58.8	14.8	59.3	60.1	14.8	61.0	62.4	14.7	63.1	<.0001
Vintage (years)	4.1	3.9	3.1	3.8	3.8	2.8	3.9	3.8	2.9	<.0001
BMI (kg/m^2^)	29.1	8.4	27.1	28.2	7.6	26.6	28.2	7.2	26.8	.0003
Hb (g/dL)	9.5	1.0	9.6	9.8	0.9	9.8	11.0	1.0	11.0	<.0001
Ferritin (ng/mL)	834	671	748	773	471	731	776	409	757	<.0001
TSAT (%)	29.0	16.7	25.0	29.1	16.5	25	31.8	13.6	29.0	<.0001
Albumin (g/dL)	3.8	0.5	3.8	3.8	0.5	3.9	4.0	0.4	4.0	<.0001
Phosphorus (mg/dL)	5.3	1.5	5.1	5.1	1.5	5.0	5.1	1.4	4.9	<.0001
Calcium (mg/dL)	9.1	0.7	9.1	9.1	0.7	9.1	9.1	0.6	9.1	<.0001
PTH (pg/mL)	561	662	387	494	478	374	442	371	354	<.0001
CRP (mg/L)	5.8	3.9	5.4	6.5	7.8	3.3	2.9	5.6	0.9	.0157
Charlson Comorbidity Index	5.2	2.0	5.0	5.3	1.9	5.0	5.5	1.9	6	<.0001

*BMI* body mass index, *CRP* C-reactive protein, *ESA*, erythropoietin-stimulating agent, *Hb* hemoglobin, *PTH* parathyroid hormone, *SD* standard deviation, *TSAT* transferrin saturation

*P* values represent a Kruskal Wallis one way analysis of variance test

**Table 3:** Comparison of baseline characteristics among ESA responders, acute hyporesponders, and chronic hyporesponders: categorical variables

	Chronic hyporesponders (*n* = 3086)		Acute hyporesponders (*n* = 6632)		ESA responders (*n* = 81,504)		*p*-value
	N	%	N	%	N	%	
Gender female	1633	52.9	3395	51.2	44,700	54.8	<0.0001
Race/ethnicity							<0.0001
American Indian	25	0.8	68	1.0	1153	1.4	
Asian	94	3.1	237	3.6	3063	3.8	
Black	1339	43.4	2806	42.3	30,309	37.2	
Hispanic	342	11.1	961	14.5	15,162	18.6	
Pacific Islander	25	0.8	49	0.7	708	0.9	
Other	204	6.6	432	6.5	4222	5.2	
White	1057	34.3	2079	31.4	26,887	33.0	
Primary cause renal disease							<0.0001
Diabetes	1182	38.0	2769	41.8	35,969	44.1	
Hypertension	901	29.2	1981	29.9	24,936	30.6	
Vascular access							<0.0001
Fistula	1726	55.9	3732	56.3	53,200	65.3	
Catheter	671	21.7	1579	23.8	11,382	14.0	
Graft	685	22.2	1321	19.9	16,904	20.7	
Other	4	0.1	0	0	17	0.02	
Diabetes	1558	50.5	3389	51.1	43,279	53.1	0.0001
Hypertension	1114	36.1	2401	36.2	28,282	34.7	0.0174
Cardiac arrest	3	0.1	7	0.1	82	0.1	0.9929
Myocardial infarction	9	0.3	13	0.2	163	0.2	0.1776
COPD	160	5.2	298	4.5	3097	3.8	<0.0001
History of GI bleed	68	2.2	73	1.1	815	1.0	<0.0001
Liver disease	71	2.3	126	1.9	1304	1.6	0.0009
HIV/AIDS	28	0.9	53	0.8	326	0.4	<0.0001
Hepatitis B	22	0.7	27	0.4	245	0.3	<0.0001
Septicemia	241	7.8	484	7.3	4646	5.7	<0.0001
Pneumonia	108	3.5	206	3.1	1875	2.3	<0.0001
Anemia hemolytic/sickle cell	68	2.2	33	0.5	245	0.3	<0.0001
Myelodysplastic syndrome	40	1.3	27	0.4	82	0.1	<0.0001
Monoclonal gammopathy	22	0.7	33	0.5	326	0.4	0.0006

*COPD* chronic obstructive pulmonary disorder, *ESA* erythropoietin-stimulating agent, *GI* gastrointestinal, *HIV/AIDS* Human Immunodeficiency Virus/Acquired Immunodeficiency Syndrome

*P*-values represent a chi square test

**Fig. 2:**
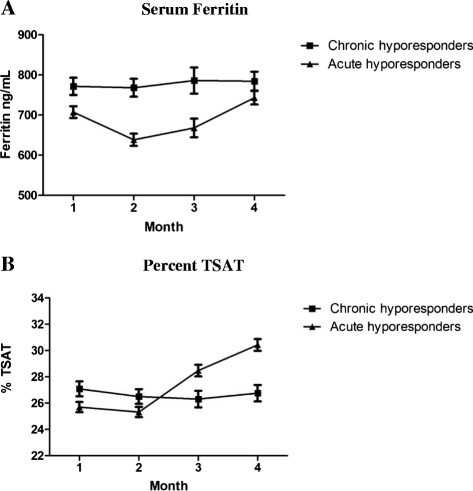
Iron storage indices are illustrated for acute and chronic ESA hyporesponsive patients over months 1 (baseline) through 4. Mean serum ferritin concentrations are shown in panel **a** (log transformed values of asymmetrically distributed ferritin values among patients). Mean percent TSAT are shown in panel **b**

In order to examine a potential role of iron deficiency in mediating ESA hyporesponse, mean TSAT and serum ferritin were studied longitudinally (Fig. 2). Over months 1 through 4, among chronic hyporesponders, mean TSAT (range 27.08 to 26.76 %) and ferritin (range 772 to 786 ng/mL) remained relatively constant (Fig. 2). For acute hyporesponders, mean TSAT increased (26.01 to 30.88 %) and mean ferritin decreased and then increased to levels greater than baseline (722 to 654 to 760 ng/mL).

**Fig. 3:**
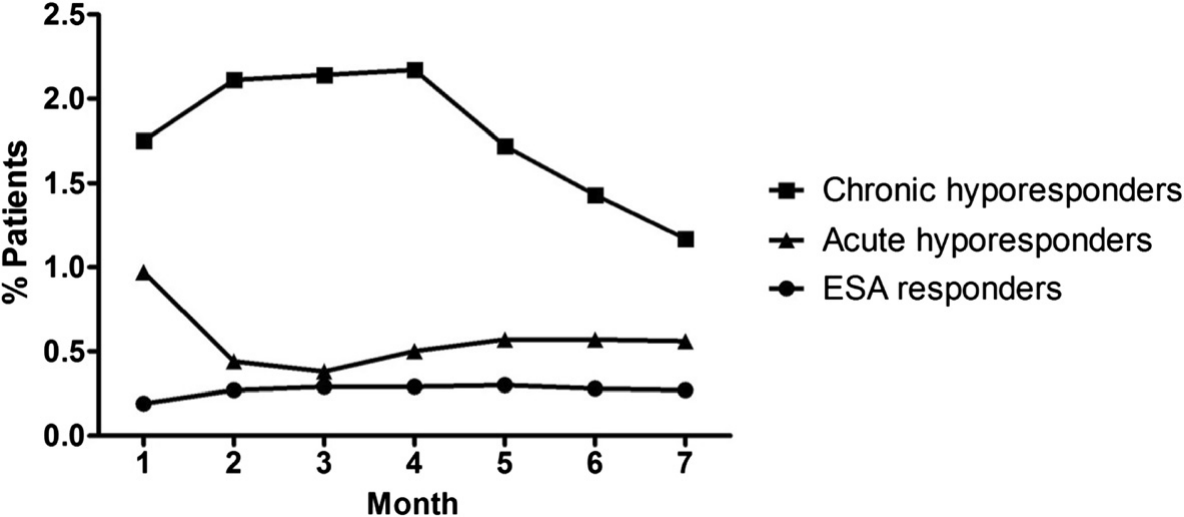
The percentage of patients who received transfusions during follow up are depicted. Results are shown for ESA responders, and acute and chronic ESA hyporesponders

Figure 3 shows the monthly frequency of transfusion among study groups over follow-up. Compared to ESA responders, on average chronic ESA hyporesponders had approximately 7-fold higher monthly burden of transfusion (range of 0.19 to 0.30 % versus 1.20 to 2.17 %, respectively). Acute hyporesponders had a 5-fold greater transfusion burden than responders initially (0.97 versus 0.19 %) that appeared to decrease with recovery of ESA responsiveness (0.56 versus 0.27 %) in later months.

Quarterly mortality data among study groups are presented in Table 4. Mortality rate was greatest in chronic ESA hyporesponders, followed by acute ESA hyporesponders, and ESA responders. On average, quarterly mortality was approximately 2.2-fold greater in chronic hyporesponders than responders, and remained persistently elevated among chronic ESA hyporesponders over the remaining quarters (range 2.98 to 5.48 %). Overall mortality was approximately 1.4-fold greater in acute hyporesponders than responders, although differences between them diminished with time (acute ESA hyporesponders range 2.17 to 3.30 %; ESA responders range 1.43 to 2.49 %).

**Table 4:** Quarterly mortality among ESA responders, acute hyporesponders and chronic hyporesponders

	Chronic Hyporesponders	Acute Hyporesponders	ESA Responders	*P* Value
Q1 (Months 5–7) % deaths	5.48 % (169/3086)	2.97 % (197/6632)	2.00 % (1631/81,504)	<0.0001
Q2 (Months 8–10) % deaths	4.56 % (125/2741)	3.06 % (189/6167)	2.16 % (1671/77,451)	<0.0001
Q3 (Months 11–13) % deaths	4.61 % (115/2497)	3.30 % (186/5723)	2.49 % (1834/73,559)	<0.0001
Q4 (Months 14, 15) % deaths	2.98 % (67/2250)	2.17 % (116/5334)	1.43 % (997/69,478)	<0.0001

Quarterly mortality was calculated by dividing the total number of deaths over the time period divided by the number of patients alive at the beginning of the time period who were not censored for discontinuation, modality change, or transplant/failed transplant. To avoid immortal time bias, mortality was considered beginning in month 5 (i.e., after the distinction between acute and chronic ESA hyporesponsiveness could be made); patients who died or were censored before 4 months had elapsed were excluded from analyses

*P* values represent a chi square test

## Discussion

The results of our analyses were used to define chronic ESA hyporesponse and differentiate it from acute hyporesponse in hemodialysis patients receiving ESA for renal anemia. Fundamental to defining chronic ESA hyporesponse was first determining what constituted ESA hyporesponse.

Studies designed to characterize or predict ESA hyporesponse have used differing methods to define it. For example, in their 10-year long study of incident hemodialysis and peritoneal dialysis patients, Suttorp et al. defined ESA resistance as hemoglobin level < 11 g/dL with an above median ESA dose depending on the method of administration (intravenous in HD patients or subcutaneous in PD patients) [23]. Others have chosen to determine ESA hyporesponse singularly by dose. In a post-hoc analysis Rossert et al. identified any patient as a hyporesponder who received subcutaneous ESA doses of > 100 IU/kg/week at any time over a 4-month dose-stabilization period [24]. Inrig et al. identified patients as ESA hyporesponders who received ≥1 μg/kg/week of darbepoetin [25]. Others have chosen to consider both patient ESA dose and hemoglobin level expressed as erythropoietin resistance index ([ERI] IU/kg/week divided by hemoglobin in g/dL) with varying ERI thresholds for analysis: Lopez-Gomez et al. prospectively stratified their analysis of factors that condition ESA response by creating ERI groups of < 5, 5–15, and > 15 [26]. In their descriptive analysis, Nishio et al. calculated the ERIs of hemodialysis patients and analyzed ESA hyporesponse by tertiles, finding that regardless of the ERI cut-off value used for study, a higher risk of death was apparent for patients falling above the chosen threshold than below it [27]. And in their meta analysis of erythropoietin resistance, authors used diverse definitions for erythropoietin resistance in patients who were not malnourished nor suffering from bleeding disorders including among others, ≥ 450 IU/kg/week intravenous or ≥ 300 IU/kg/week subcutaneous erythropoietin, and ≥ 1.5 μg/kg/week of darbepoetin [28].

In our analysis, we defined chronic ESA hyporesponse and attempted to differentiate it from acute hyporesponse taking into consideration both hemoglobin level and ESA dose. Criteria for chronic hyporesponse must consider the dramatic evolution of anemia management that has occurred over recent years in the United States [20, 22]. Here we identified a series of contiguous ESA dose-by-hemoglobin strata that are logically consistent with ESA hyporesponse and ESA dosing distributions in hemodialysis patients. Our findings are strengthened by the fact that the proportion of patients identified as ESA hyporesponsive was similar prior to (July 2011) and following the recent changes in ESA dosing patterns (February 2012) [20, 22]. Building on these criteria for hyporesponse, we observed that most patients who were identified as hyporesponsive at baseline (month 1) but went on to recover ESA responsiveness, did so by month 4. Results from our Kaplan Meier analysis demonstrated that after more than 4 months of meeting the baseline definition of ESA hyporesponsiveness subsequent recovery of ESA responsiveness was less likely, thereby providing an empiric based definition of chronic ESA hyporesponsiveness.

Recently, Gilbertson et al. proposed a definition of chronic ESA hyporesponse of ≥ 4 consecutive months of elevated ESA dose greater than the ninetieth percentile (176,400 IU monthly, approximately 13,500 U/treatment) [11]. It is reassuring that when methodologies different than ours were used to identify ESA hyporesponse, 4 months was determined as the requisite period necessary to characterize it as chronic. However, the Gilbertson et al. study was conducted with hemodialysis patient data generated during 2008, and the ESA dose criteria do not reflect changes in ESA dosing that have occurred since 2011.

The many medical conditions that contribute to anemia in patients with chronic kidney disease are probably the same factors that determine the nature of ESA hyporesponse [19], such as inflammation [7, 12, 25], prevalent comorbidities [7, 26], and medications [29], as well as iron deficiency [29, 30]. Iron deficiency may influence ESA hyporesponse in some dialysis patients, and has been associated with reduced TSAT in patients with hyporesponse [26, 29–31]. However, we did not find evidence of widespread inadequate iron stores among ESA hyporesponsive patients [7]. In our study, mean ferritin levels were > 650 ng/mL in both acute and chronic ESA hyporesponders. Among acute hyporesponders, iron deficiency may have played somewhat of a mediating role: TSAT levels rose concurrently with restitution of ESA responsiveness. Conversely, among chronic ESA hyporesponders, TSAT levels were constant over time, and in excess of 20 %, not suggestive of relative iron deficiency in this group.

Decades after the introduction of ESAs to treat anemia of renal disease, the rationale for transfusion avoidance is still valid [32], but for patients suffering from ESA hyporesponse, our data demonstrate that red cell transfusion may become an undesirable necessity [15]. Of great importance is the finding that transfusion burden was distinct among ESA responsive, acute hyporesponsive, and chronic hyporesponsive patients. There was a graded transfusion risk among the 3 patient groups, with the greatest in patients who would go on to become categorized as chronic hyporesponders and lowest in ESA responsive patients. Moreover, whereas the difference between acute hyporesponders and responders in frequency of transfusion diminished over time, the frequency of transfused patients among chronic hyporesponders remained persistently higher than the other groups.

Low hemoglobin concentration [6, 33–35] and high erythropoietin requirement has been associated with mortality in patients with renal anemia [6, 23, 26, 30]. Our study findings also indicated a graded pattern of mortality, with chronically hyporesponsive patients having greater mortality rates than acutely hyporesponsive patients, each of which had greater mortality rates than patients with ESA responsiveness. The higher rate of mortality for patients with chronic ESA hyporesponsiveness persisted for at least 9 months, well beyond the requisite 4-month period over which chronic hyporesponsiveness was defined. By contrast, mortality differences between acute hyporesponsive patients and ESA responsive patients abated with time.

A number of limitations inherent to observational research apply to our analysis. Over the 15-month study period it is possible that new illnesses emerged that were not identified at the baseline month, potentially confounding results. Another limitation is that all data were taken from a single dialysis provider organization, and its clinical patient database may have been missing some patient history and prior diagnoses information. Furthermore, we did not exclude conditions that are likely to be associated with hyporesponsiveness, e.g., gastrointestinal bleeds (a major cause of hyporesponsiveness) and patients with myelodysplastic syndrome. However, these conditions as captured in our database are exceedingly rare. Therefore generalization of our study findings to all hemodialysis patient populations is not guaranteed. However, this concern is allayed somewhat given that this provider cares for approximately one third of all hemodialysis patients in the United States, and that our results are in agreement with other research around chronic ESA hyporesponse [11].

## Conclusions

Our study findings demonstrate that chronic ESA hyporesponse is distinct from acute hyporesponse and can be defined using hemoglobin levels and ESA dose as hyporesponse that persists for at least 4 months. Chronic ESA hyporesponsive patients have persistently greater burdens of transfusion and death compared to other patients. Additional studies are needed to further validate our definitions and to test their generalizability to other hemodialysis populations.

## Additional file

Additional file 1: Table S1. Baseline Distribution of ESA Dose by Hemoglobin Concentration: July 2011. (DOC 54 kb)

## Abbreviations

BMI: Body mass index
COPD: Chronic obstructive pulmonary disorder
CRP: C-reactive protein
ERI: Erythropoietin resistance index
ESA: Erythropoietin-stimulating agent
ESRD: End Stage Renal Disease
GI: Gastrointestinal
Hb: Hemoglobin
HIV/AIDS: Human Immunodeficiency Virus/Acquired Immunodeficiency Syndrome
KDIGO: Kidney Disease Improving Global Outcome
PTH: Parathyroid hormone
SD: Standard deviation
TSAT: Transferrin saturation
