# Supplementary material for: Characterization of chronic and acute ESA hyporesponse: a retrospective cohort study of hemodialysis patients
Source: BMC Nephrol. 2015 Aug 18;16:144. doi: 10.1186/s12882-015-0138-x (PMC4539683; doi:10.1186/s12882-015-0138-x)
Supplement: Additional file 1: Table S1. — Baseline Distribution of ESA Dose by Hemoglobin Concentration: July 2011. (DOC 54 kb) [file 12882_2015_138_MOESM1_ESM.doc]

**Additional file 1: Table S1. Baseline Distribution of ESA Dose by Hemoglobin Concentration: July 2011**

|  | | ESA Dose Per Session (IU) Range | | | | | | | | | |  |
| --- | --- | --- | --- | --- | --- | --- | --- | --- | --- | --- | --- | --- |
|  |  | 0 | 0- 2,000 | > 2,000-  4,000 | > 4,000-  6,000 | > 6,000-  8,000 | > 8,000-10,000 | > 10,000-12,000 | > 12,000-14,000 | > 14,000-16,000 | > 16,000 |  |
|  |  | % (n) | % (n) | % (n) | % (n) | % (n) | % (n) | % (n) | % (n) | % (n) | % (n) | % (n) |
| **Missing** | | 0.31 (285) | 0.07 (66) | 0.17 (162) | 0.12 (114) | 0.08 (72) | 0.07 (67) | 0.03 (31) | 0.02 (21) | 0.03 (27) | 0.09 (81) | 0.99  (926) |
| **Hemoglobin Range (g/dL)** | ≤8 | 0.02 (23) | 0.01 (8) | 0.01 (12) | 0.02 (19) | 0.03 (25) | 0.02 (16) | 0.02 (21) | 0.02 (14) | 0.02 (20) | 0.19 (175) | 0.36  (333) |
|  | >8-8.5 | 0.03 (30) | 0.01 (5) | 0.03 (28) | 0.05 (46) | 0.04 (37) | 0.04 (34) | 0.02 (19) | 0.03 (30) | 0.02 (16) | 0.19 (174) | 0.45  (419) |
|  | >8.5-9 | 0.05 (43) | 0.02 (21) | 0.06 (52) | 0.11 (100) | 0.1 (95) | 0.1 (89) | 0.07 (63) | 0.07 (62) | 0.06 (52) | 0.33 (311) | 0.95  (888) |
|  | >9-9.5 | 0.06 (60) | 0.05 (49) | 0.18 (165) | 0.22 (206) | 0.22 (204) | 0.19 (174) | 0.14 (127) | 0.1 (92) | 0.09 (80) | 0.54 (503) | 1.78  (1,660) |
|  | >9.5-10 | 0.12 (115) | 0.18 (170) | 0.51 (478) | 0.69 (640) | 0.51 (471) | 0.41 (381) | 0.28 (261) | 0.21 (200) | 0.2 (184) | 0.73 (680) | 3.84  (3,580) |
|  | >10-10.5 | 0.21 (199) | 0.53 (492) | 1.79 (1,671) | 1.75 (1,634) | 1.2 (1,116) | 0.77 (714) | 0.53 (493) | 0.33 (310) | 0.36 (338) | 0.73 (681) | 8.2  (7,648) |
|  | >10.5-11 | 0.38 (351) | 2.4 (2,240) | 4.93 (4,592) | 3.3 (3,075) | 1.78 (1,659) | 1.17 (1,093) | 0.72 (669) | 0.45 (423) | 0.41 (386) | 0.55 (513) | 16.09  (15,001) |
|  | >11-11.5 | 0.6 (556) | 7.02 (6,545) | 7.76 (7,232) | 3.65 (3,406) | 1.91 (1,784) | 1.36 (1,264) | 0.67 (621) | 0.46 (428) | 0.32 (294) | 0.29 (270) | 24.03  (22,400) |
|  | >11.5-12 | 1.17 (1,089) | 10.01 (9,328) | 5.76 (5,370) | 2.62 (2,439) | 1.45 (1,350) | 0.88 (819) | 0.47 (440) | 0.26 (239) | 0.11 (104) | 0.14 (134) | 22.87 (21312) |
|  | >12 | 7.06 (6,577) | 7.18 (6,691) | 3.26 (3,042) | 1.48 (1,377) | 0.72 (674) | 0.37 (342) | 0.17 (163) | 0.09 (82) | 0.04 (37) | 0.05 (48) | 20.42 (19,033) |
|  | | 10.01 (9,328) | 27.48  (25,615) | 24.47  (22,804) | 14.01  (13,056) | 8.04  (7,487) | 5.36  (4,993) | 3.12  (2,908) | 2.04  (1,901) | 1.65  (1,538) | 3.83  (3,570) | 100  (93,200) |
| Shaded cells indicate domains consistent with the baseline definition of ESA hyporesponse. | | | | | | | | | | | | |
